# Supplementary material for: What makes a temperate phage an effective bacterial weapon?
Source: mSystems. 2024 May 10;9(6):e01036-23. doi: 10.1128/msystems.01036-23 (PMC11237456; doi:10.1128/msystems.01036-23)
Supplement: Supplemental material — Fig. S1 to S10. [file msystems.01036-23-s0001.pdf]

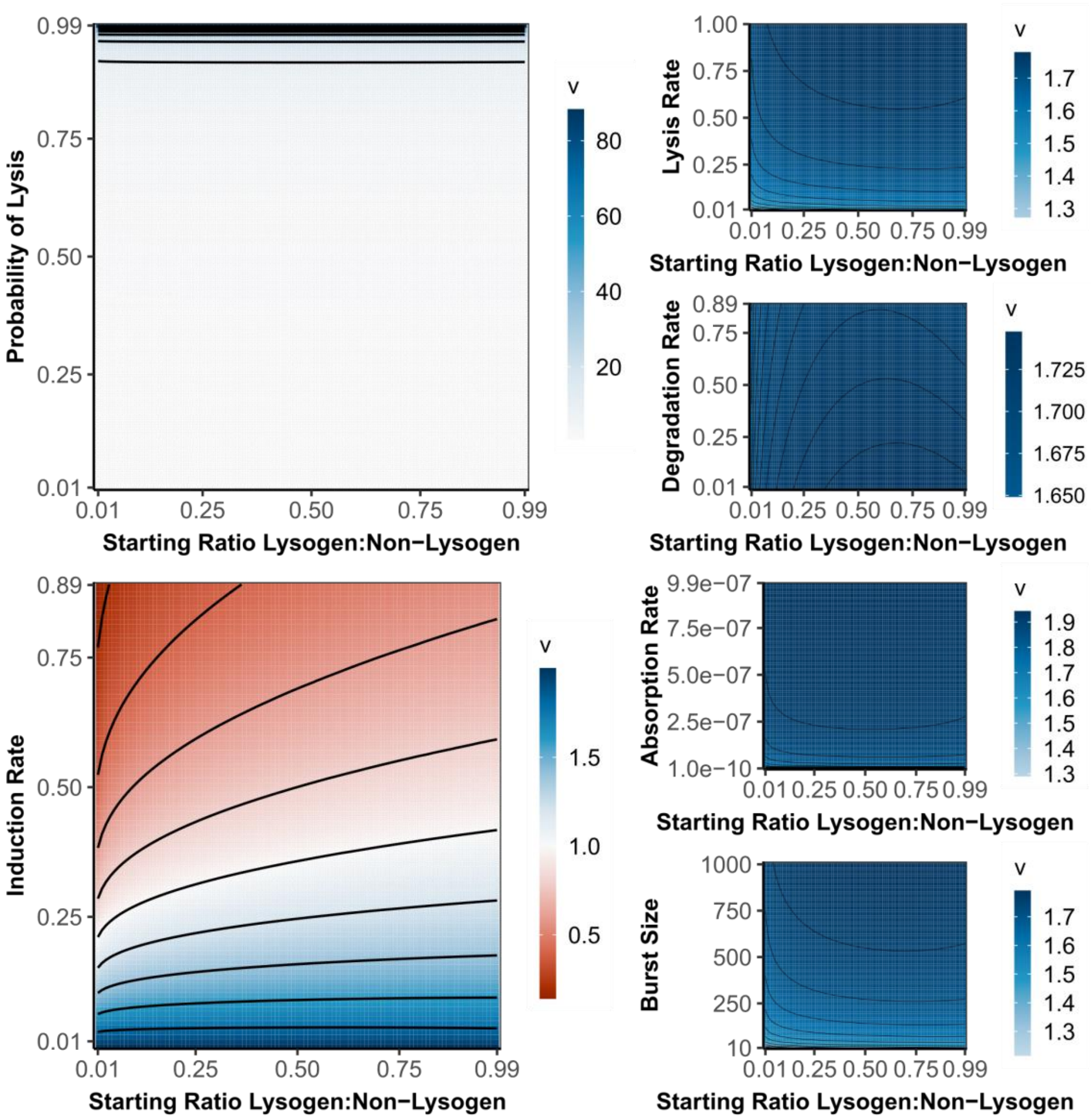

SI 1. Analysing the impact of life history traits and frequency when phage effectiveness is calculated as the relative fitness measure, *v*, of the lysogen. Here each heatmap is coloured by the relative fitness of the focal strain compared to the background strain, given by the equation:

$$v = \frac{F_2 * (1 - F_1)}{F_1 * (1 - F_2)}$$

Where F_1 and F_2 represent the starting and final frequencies of the Focal strain respectively. As in our original analyses, we find that probability of lysis and induction rate have the greatest impact on PEI, that PEI varies with lysogen starting frequency, and that the interplay between life history traits (LHT) and frequency leads to cases where a phage is beneficial under some

conditions but detrimental under others. However, as relative fitness tends to saturate quickly these effects are less prominent than with other measures.

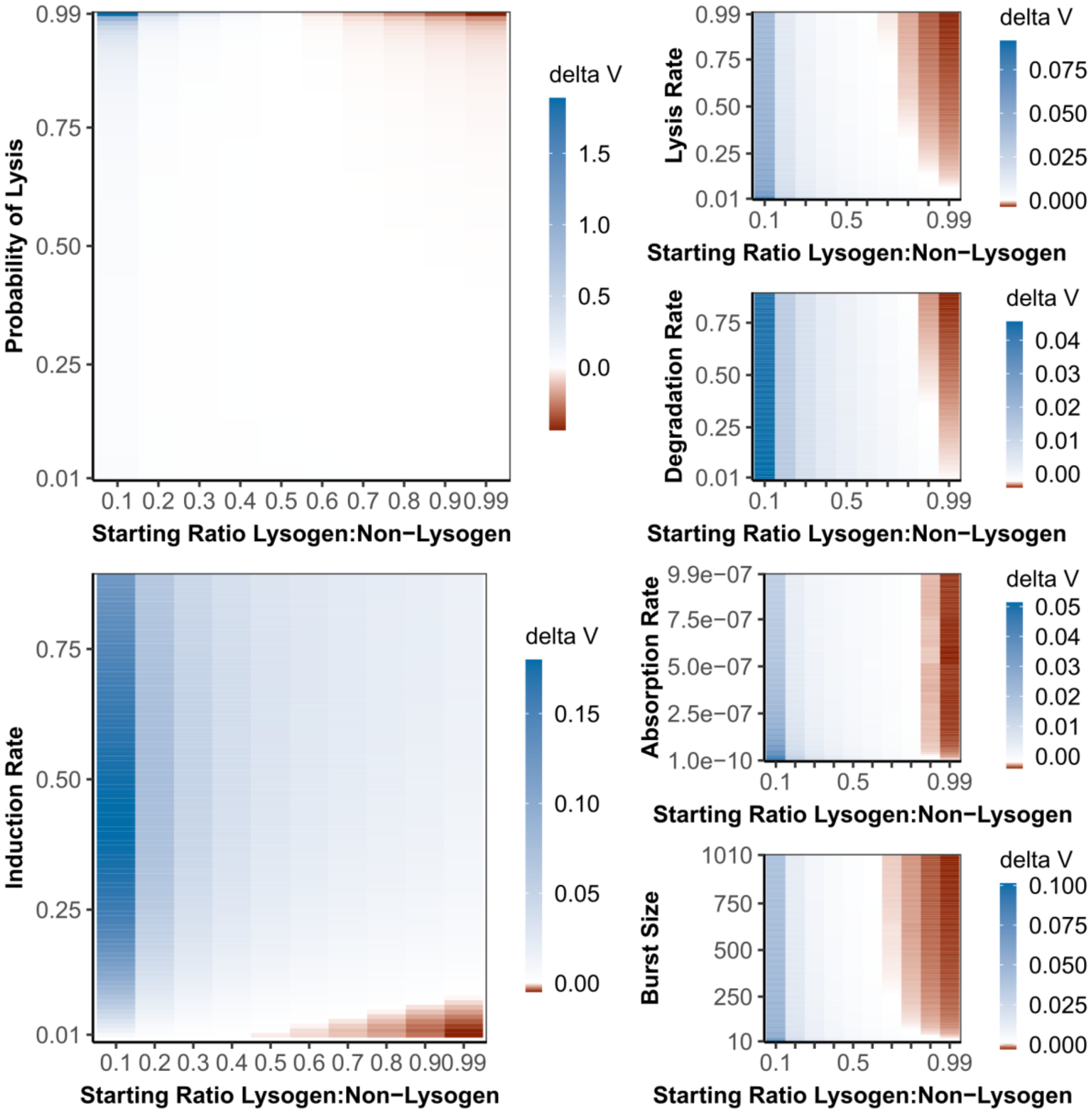

SI 2. Confirming the frequency dependence of PEI when using relative fitness. When calcuting PEI as the relative fitness of the focal strain compared to the background strain, changes with frequency are small and thus challenging to detect via eye. To confirm the dependence of PEI on frequency we therefore also plot the derivative of PEI against starting frequency (i.e. $\Delta v = \mathrm{dPEI}/\mathrm{dFrequency}$). Here $\Delta v > 0$ indicates that PEI increases with increasing starting frequency, while $\Delta v < 0$ indicates that PEI decreases with increasing

starting frequency. For all life history trait analyses, we see that the focal strains relative frequency peaks at intermediate starting frequencies.

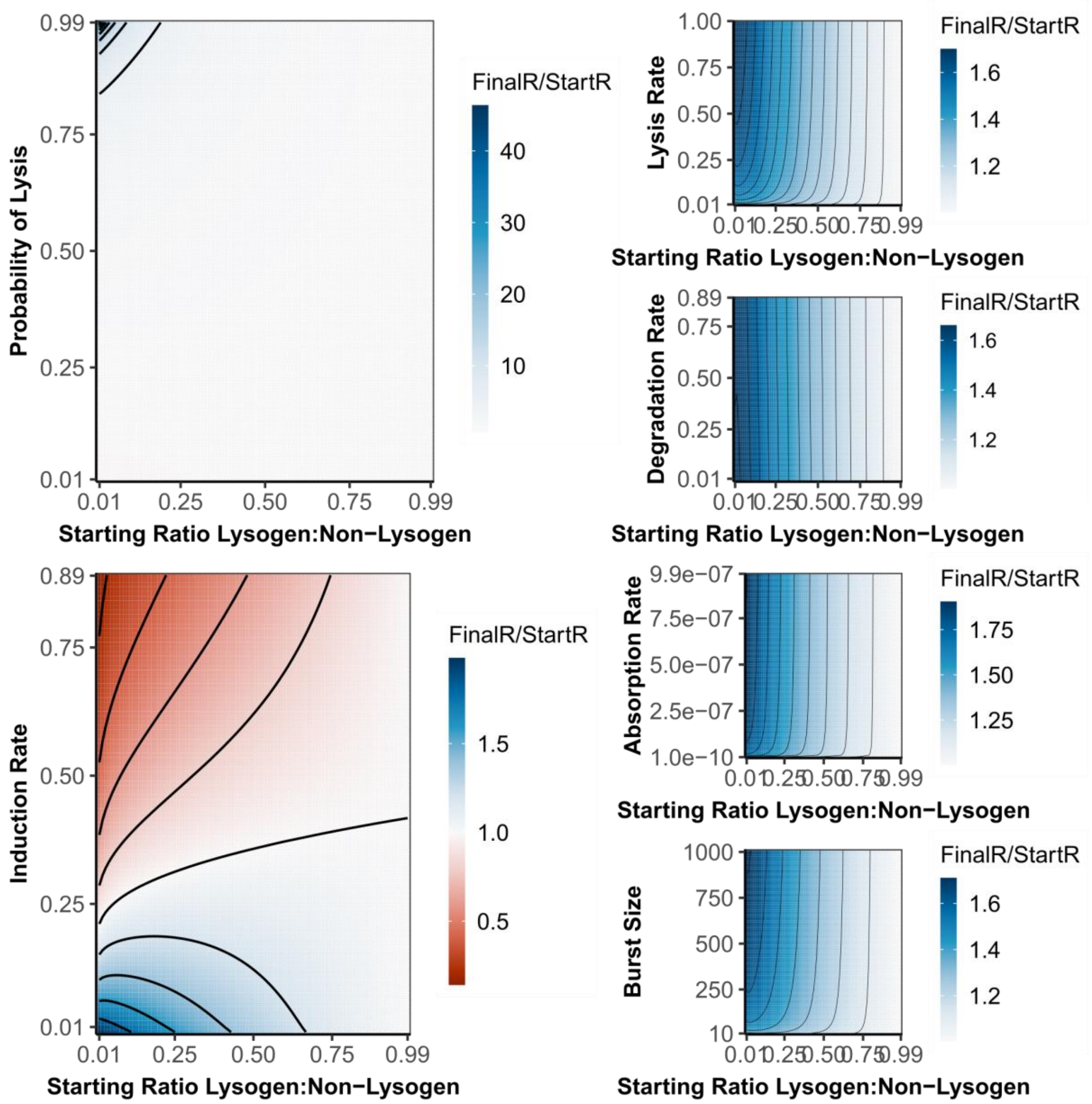

SI 3. Analysing the impact of life history traits (LHT) and frequency when phage effectiveness is calculated as the invasion speed of the focal strain. Each heatmap is the same simulations from Figure 2 but the colour now shows the PEI calculated as the natural log of the final frequency of the Focal lysogen (F) divided by the starting frequency. As in our original analyses, we find that probability of lysis and induction rate have the greatest impact on PEI, that PEI varies with lysogen starting frequency, and that the interplay between LHT and frequency leads to cases where a phage is beneficial under some conditions but

detrimental under others. However, under this measure PEI is often (although not always) greatest when lysogens are rare, as this measure of competitiveness highlights relatively small changes by initially rare species.

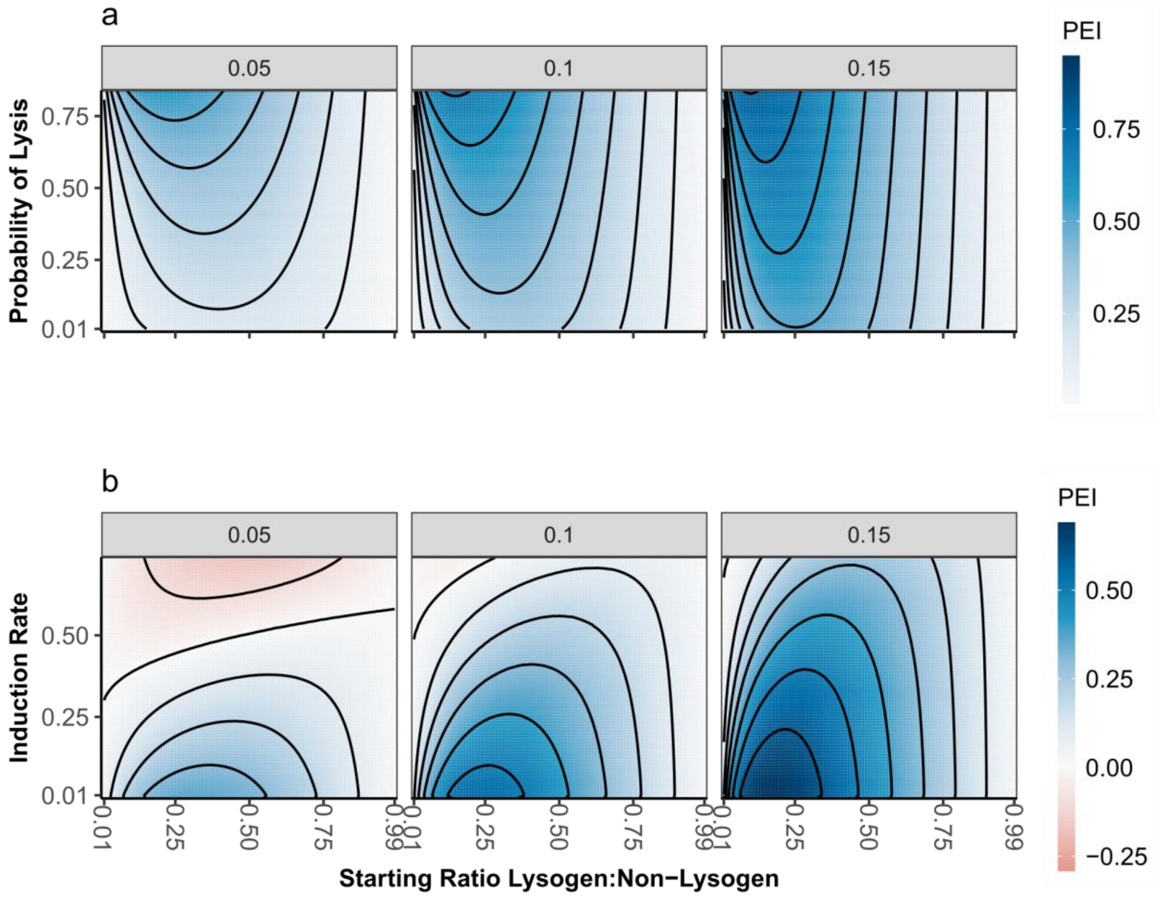

SI 4. Phage weapons are more useful when novel competitor lysogens have a higher induction rate than Focal strain lysogens. Newly generated lysogens are observed to have higher induction rates than more established lineages, prompting us to investigate the effect of induction rate assymetry on PEI. In all tested conditions a higher induction rate in the novel lysogen amplified the benefit of the phage. This amplified benefit can compensate for the otherwise detrimental effect of high induction rates in the focal strain, such that even phage with very high induction rates can be benefical. Rows correspond to the relationship between

probability of lysis (a) or induction rate (b) and PEI in three different conditions. The number above each plot shows the factor the induction rate of the novel lysogen is increased by (Novel lysogen induction rate = Focal induction Rate or PoL + Number).

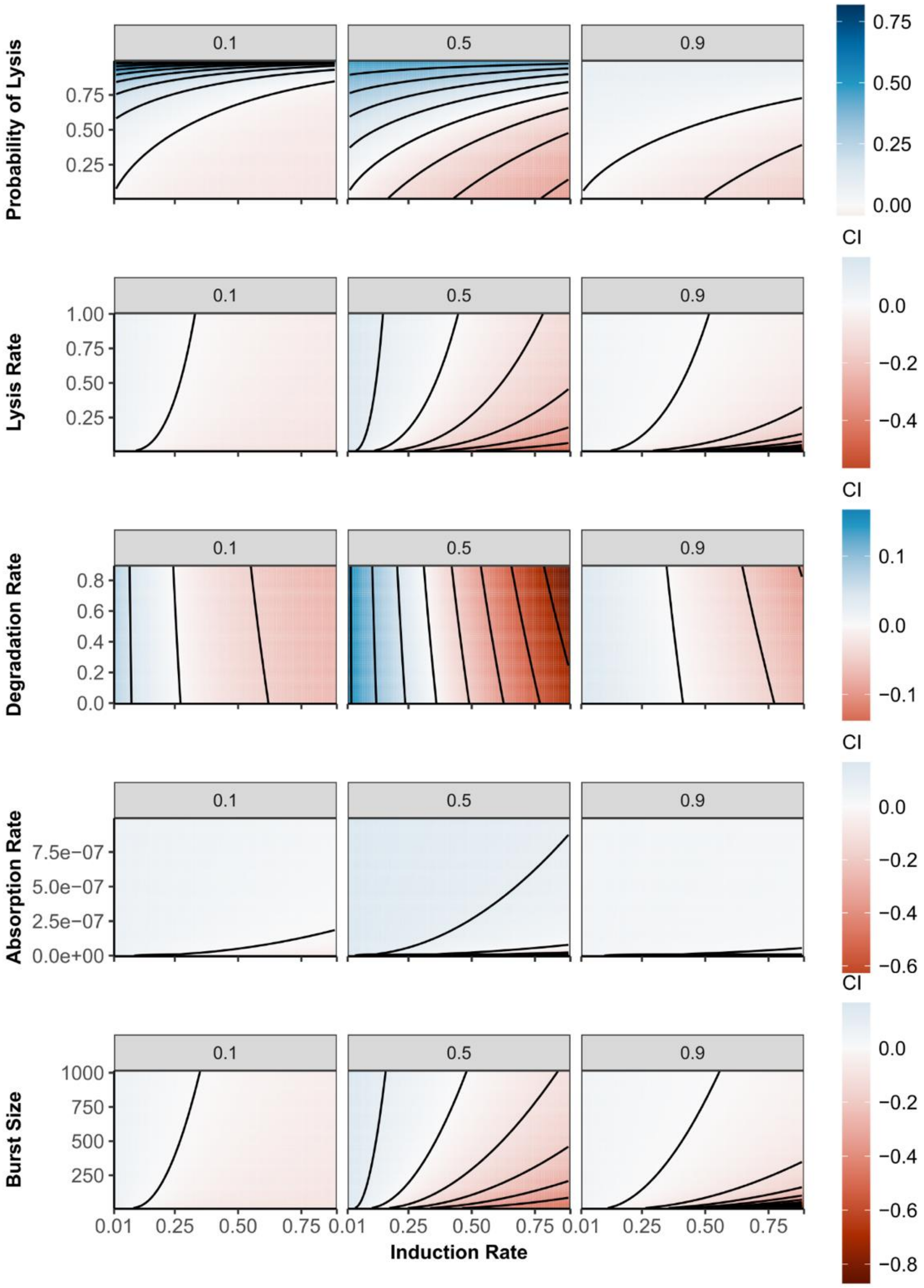

SI 5. Compensatory effects of life history traits are observed at several starting frequencies. Each heatmap shows equivalent parameter sweeps to those performed in Fig.3,

but with different starting frequencies of the Focal strain. While at different frequencies we see subtle changes in the dynamics, the overall relationship remains the same.

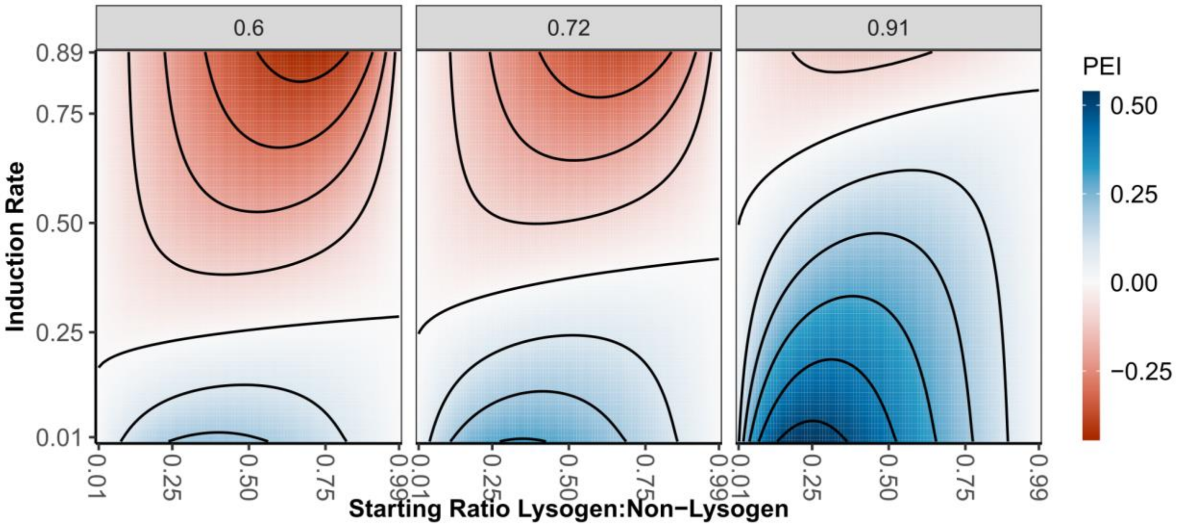

SI 6. Using experimentally determined probability of lysis values does not alter the relationship between induction rate, initial frequency, and PEI. Our initial parameter sweeps used a relatively low value for probability of lysis compared with those observed in our experiments, thus here we repeat our Figure 1 analyses using these higher probability of lysis values.

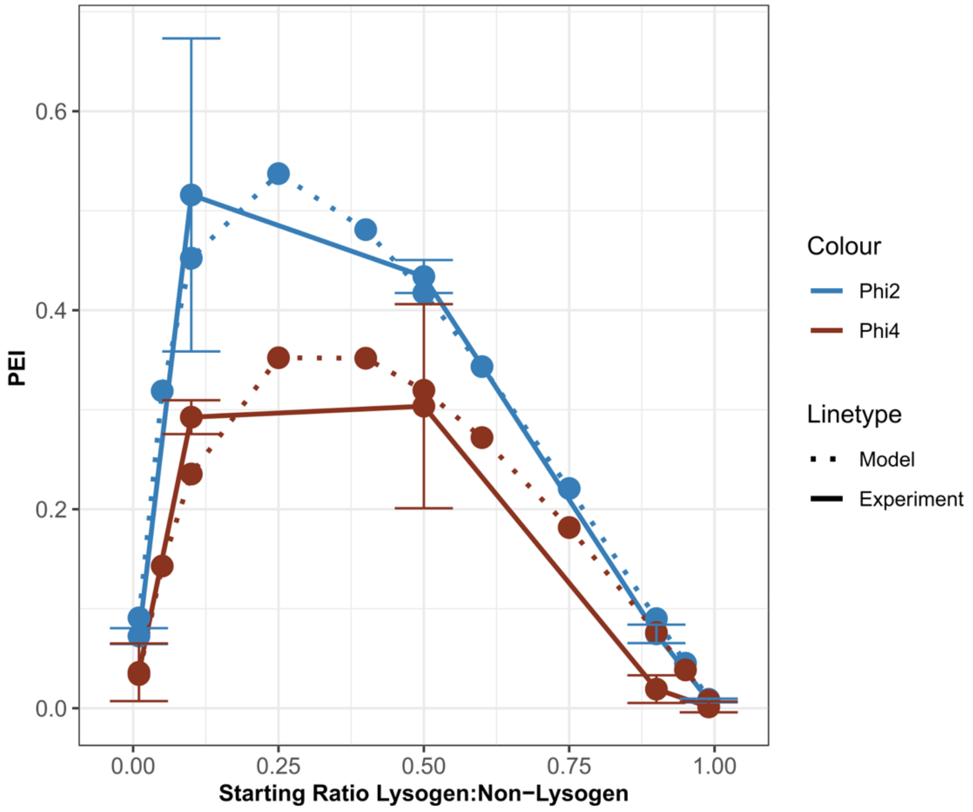

SI 7. Our nutrient-explicit model can also well accurately predict our experimental results. Predicted values for the nutrient model are shown in dashed lines while experimental values are shown with solid lines. The predictions for Phi2 are shown in blue and Phi4 in red. The nutrient model also predicted Phi2 to be more competitive than Phi4 while also recapitulating the shape of the curve.

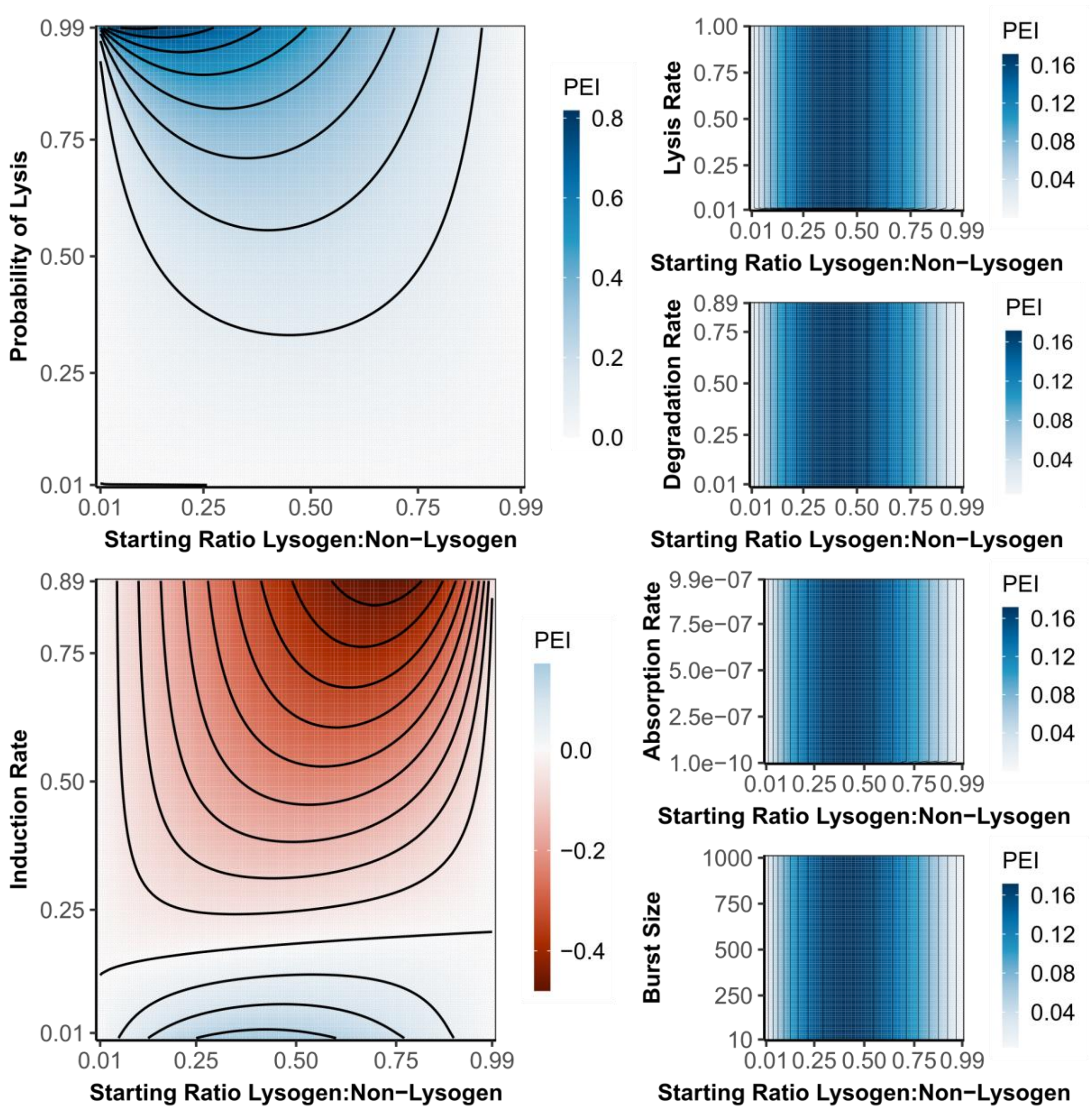

SI 8. The nutrient model also recreates the relationship between different life history traits and starting frequency seen in figure 2. As before probability of lysis and induction rate have the largest effect on the competitiveness of the lysogens. While the dynamics between the models are subtly different, the overall relationships between the traits and starting frequency remains the same.

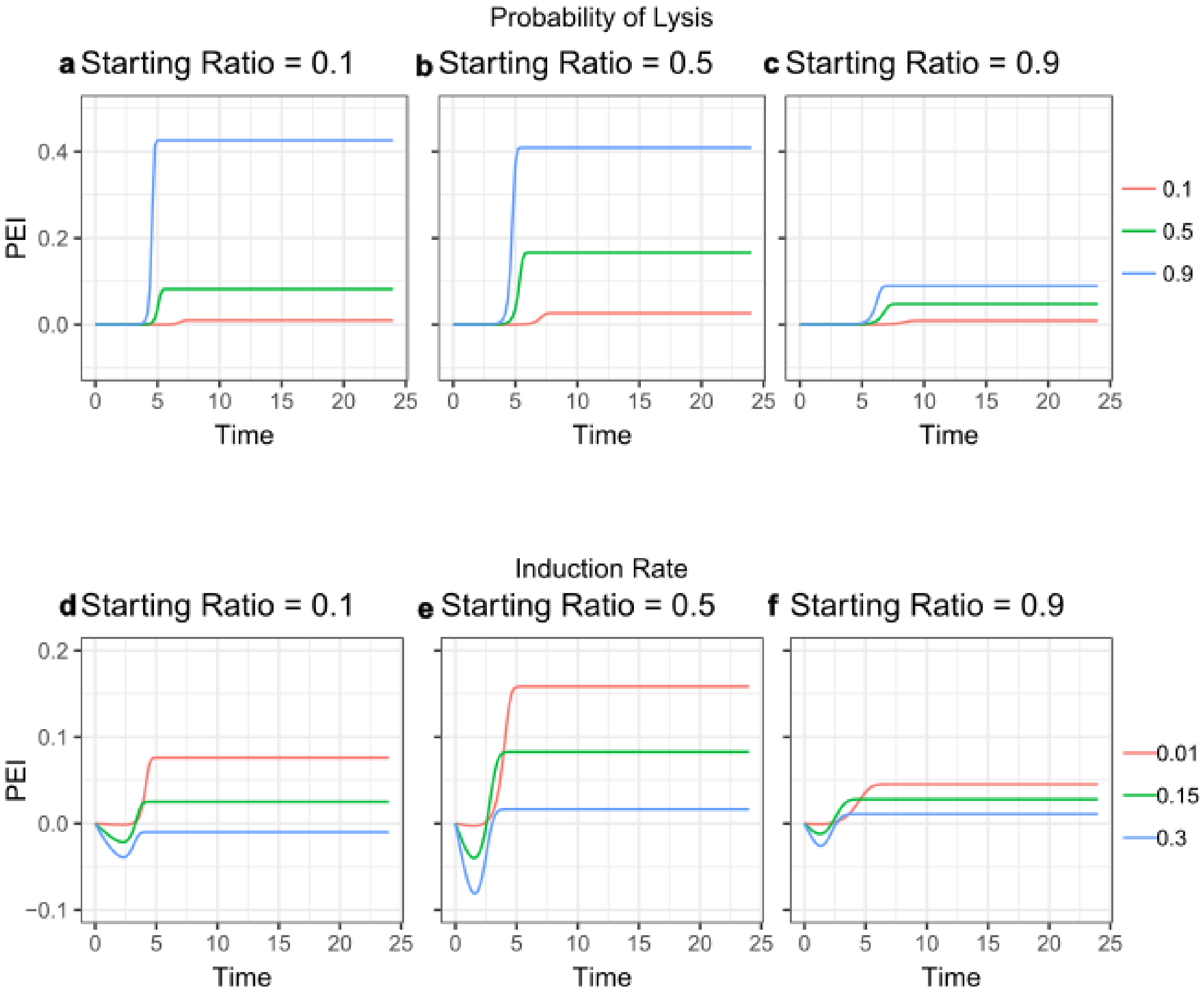

SI 9. PEI varies over time, but relationships between PEI and individual life history traits remain consistent. a-c Changes in PEI over time for three different phage, varying in their probability of lysis (π, colours) under three different starting ratios. In these cases PEI monotonically increases over time as the phage spreads within the system, and the higher the probability of lysis, the higher the PEI. **d-f** Changes in PEI over time for three different phage, varying in their induction rate (κ, colours) under three different starting ratios. When induction rates are high the phage is initially highly detrimental, however this cost lessens over time as the phage spreads within the system and passes on the cost of the phage to the novel lysogens. Notably this can result in the phage being costly or beneficial depending on how

long the competition is observed, and can even lead to scenarios where the optimal phage weapon switches over time.

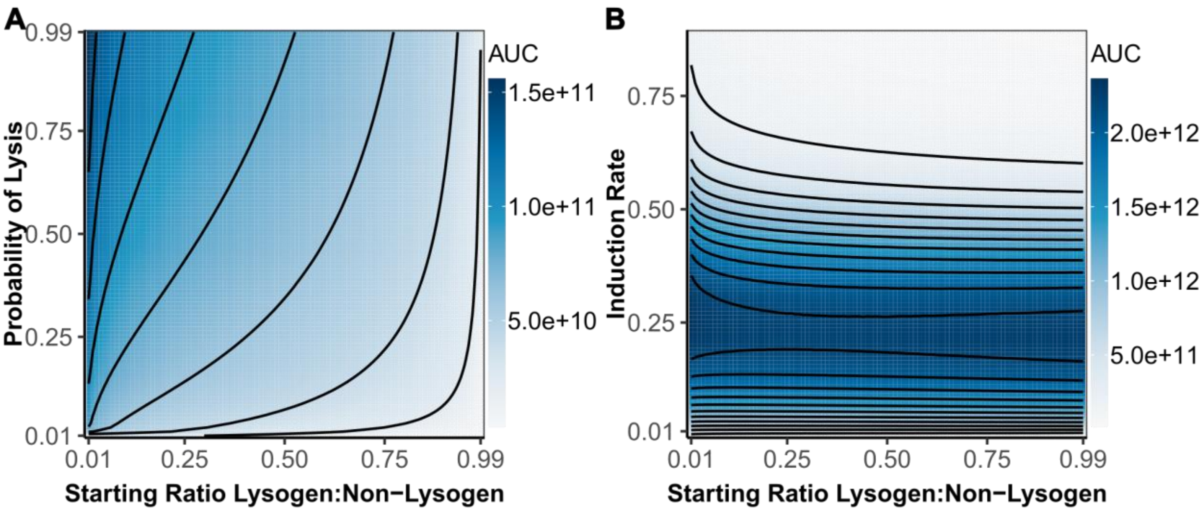

SI 10. The total amount of phage produced during a competition is dependent on the starting frequency of lysogens and life history traits. A. Plotting how total phage virions produced (calculated as the integral of phage abundance over time) changes depending upon initial lysogen frequency and probability of lysis. The higher the probability of lysis, the more phage virions produced. B. Plotting how total phage virions produced changes depending upon initial lysogen frequency and induction rate. Here phage production peaks at intermediate induction rates.
